# Two novel multiple endocrine neoplasia type 1 variants caused thymic neuroendocrine tumor: a case report

**DOI:** 10.1186/s13053-026-00331-4

**Published:** 2026-02-24

**Authors:** Zi Dai, Jing Zhang, Jing Wang, Leshi Ma, Pei Liao, Xiaojie Deng, Zhenxiang Li, Zhijie Luo, Jieshan Guan

**Affiliations:** 1 The First Clinical Medical College, Guangzhou University of Chinese Medicine, Sanyuanli Campus, No.12, Jichang Road, Baiyun District, Guangzhou, Guangdong 510405 P. R. China; 2 Shenshan Hospital, The First Affiliated Hospital of Guangzhou University of Chinese Medicine, No. 16, Kangping Road, Dongxing Community, Xiangzhou Street, Shanwei City, Guangdong 516600 P. R. China; 3 Department of Medical Oncology, The First Affiliated Hospital of Zhejiang Chinese Medical University, Zhejiang Provincial Hospital of Traditional Chinese Medicine, Hangzhou, 310053 P. R. China; 4 The First Affiliated Hospital of Guangzhou, Chongqing Hospital, University of Chinese Medicine, No.380, Jiagjun Road, Beibei District, Chongqing City, 400700 P. R. China; 5 Department of Pulmonary and Critical Care Medicine, The Second Affiliated Hospital of Guangzhou Medical University, No. 250 Cheung Kong East Road, Haizhu District, Guangzhou, Guangdong P. R. China; 6 Cancer Center, The First Affiliated Hospital of Guangzhou, University of Chinese Medicine, No.12, Jichang Road, Baiyun District, Guangzhou, Guangdong 510405 P. R. China; 7 Guangdong Clinical Research Academy of Chinese Medicine, Guangzhou, Guangdong 510405 P. R. China; 8 Lingnan Medical Research Center, Guangzhou University of Chinese Medicine, No.12, Jichang Road, Baiyun District, Guangzhou, Guangdong 510405 P. R. China

**Keywords:** Thymic neuroendocrine tumors, Multiple endocrine neoplasia type 1, Two-hit hypothesis, Whole exome sequencing, Case report

## Abstract

Multiple endocrine neoplasia type 1 (MEN1), or Wermer’s syndrome, is a rare autosomal dominant genetic disorder caused by *MEN1* mutations, which rarely result in thymic tumors. However, effective therapies or standard treatments are still lacking for patients with MEN1-associated tumors. Some patients with MEN1-associated tumors, such as parathyroid carcinoma and insulinoma, have both germline and somatic *MEN1* mutations, consistent with Knudson’s two-hit hypothesis. However, this hypothesis has seldom been reported in connection with MEN1-associated thymic tumors. Herein, we observed a family carrying the *MEN1* p.L105Sfs*14 mutation in which two males were diagnosed with MEN1-associated thymic neuroendocrine tumors (NETs). The proband was diagnosed with a left adrenal cortical adenoma and underwent surgery at 49 years of age. After two years, he underwent another surgery for thymic NET. The proband’s son underwent robot-assisted resection at the age of 29 years. Whole exome sequencing (WES) and 425 cancer-related gene panel sequencing revealed that they both had the same germline *MEN1* p.L105Sfs*14 mutation and that the son carried the somatic *MEN1* p.Q96* mutation. We present a case of two novel *MEN1* variants (p.L105Sfs*14 and p.Q96*) in thymic NET. These mutations have not been previously reported in patients with MEN1-associated thymic NET; they resulted in the production of a truncated menin protein. The two-hit of *MEN1* germline and somatic mutations occurred in the thymic tumor of the proband’s son. This may partially be the reason for early tumor onset and is consistent with the “two-hit hypothesis”.

## Introduction

Multiple endocrine neoplasia type 1 (MEN1), or Wermer’s syndrome, is caused by the inactivation of the tumor-suppressing gene *MEN1*; it is characterized by multiple endocrine tumors, with an estimated prevalence of 1–10 per 100,000 people [1]. It is an autosomal dominant disorder that may induce tumor development in the parathyroid glands, anterior pituitary, and endocrine cells of the pancreas [2, 3].

*MEN1* is located on chromosome 11q13 and comprises 10 exons that encode a 610-amino acid (AA) protein called menin [1, 4]. Emerging evidence suggests that menin, a nuclear scaffold protein, plays an important role in coordinating chromatin remodeling, genome stability, and regulating gene transcription, cell proliferation, and apoptosis [5–7]. Over 70% of *MEN1* mutations are predicted to lead to the expression of truncated forms of menin [8]. According to the three-dimensional (3D) structure, menin is a single-domain protein, and truncation of its AA sequence will lead to the destruction of protein folding and loss of function [9].

According to Knudson’s two-hit hypothesis [3], at least two variants or hits are required for cells to become cancerous, which is always accompanied by loss of heterozygosity (LOH) [9]. Most MEN1-associated tumors exhibit biallelic inactivation of the suppressor gene owing to somatic loss of the wild-type (WT) allele, resulting in somatic LOH [10]. Studies have reported that only 25% of thymic neuroendocrine tumors (NETs) are associated with MEN1 [11], and the LOH in MEN1-associated thymic tumors remains controversial [10]. Thymic NETs are extremely rare but aggressive, accounting for approximately 5% of all tumors in the thymus and mediastinum, and have an estimated incidence of 1 per 5 million people [12–14].

Herein, we present a case of two novel *MEN1* variants (p.L105Sfs*14 and p.Q96*), which have not been reported in thymic NETs. Further, we summarize the results of 3D protein structure simulations and hypothesize the structures of the pretruncated menin protein. We present the following case in accordance with the CARE reporting checklist.

## Method

The bioinformatics characteristics of *MEN1* gene mutations in cancer and their impact on protein function were explored through public databases and computer simulation technology. We performed a combined analysis of 10 pan-cancer studies using an online open database, cBioPortal (https://www.cbioportal.org) [15, 16] and prepared the OncoPrint map and lollipop chart to present the clinical features of the patients with *MEN1* mutations and specific menin-related mutation information respectively.

The corresponding AA sequences of the relevant proteins were searched on the National Center for Biotechnology Information (NCBI) website (https://www.ncbi.nlm.nih.gov/) and then aligned using ClusatlW. The conservatism of the AAs affected by the *MEN1* p.L105Sfs*14(c.313del) and p.Q96*(c.286 C > T) variants was analyzed using ClusatlW [17] with default parameters.

Bioinformatics tools were used to predict and compare the structures of wild-type and mutant *MEN1* proteins to explore the effect of mutations on protein function. The AA sequences were obtained from the UniProt database [18, 19] (https://www.uniprot.org/, entry identifier: O00255), and the structure of human WT crystallin gamma D was obtained from the Protein Data Bank (PDB) database (ID:3U84). The artificial intelligence software AlphaFold2 [20] was used to predict structural changes and visualize the protein structure. Because the homologous structure of 3U84 was missing a part of the flexible loop, Alphafold2 was used to predict the models of the intact proteins. Finally, the predicted protein structure of WT *MEN1* was obtained. The mutation sites were analyzed to obtain the mutant sequences, including *MEN1* p.L105Sfs*14 and p.Q96*. Thereafter, using 3U84 as the protein structure model and SWISS-MODEL (https://swissmodel.expasy.org/) [21], the protein models were predicted, and PyMoL was used to visualize the protein structures.

## Case presentation

**Fig. 1:**
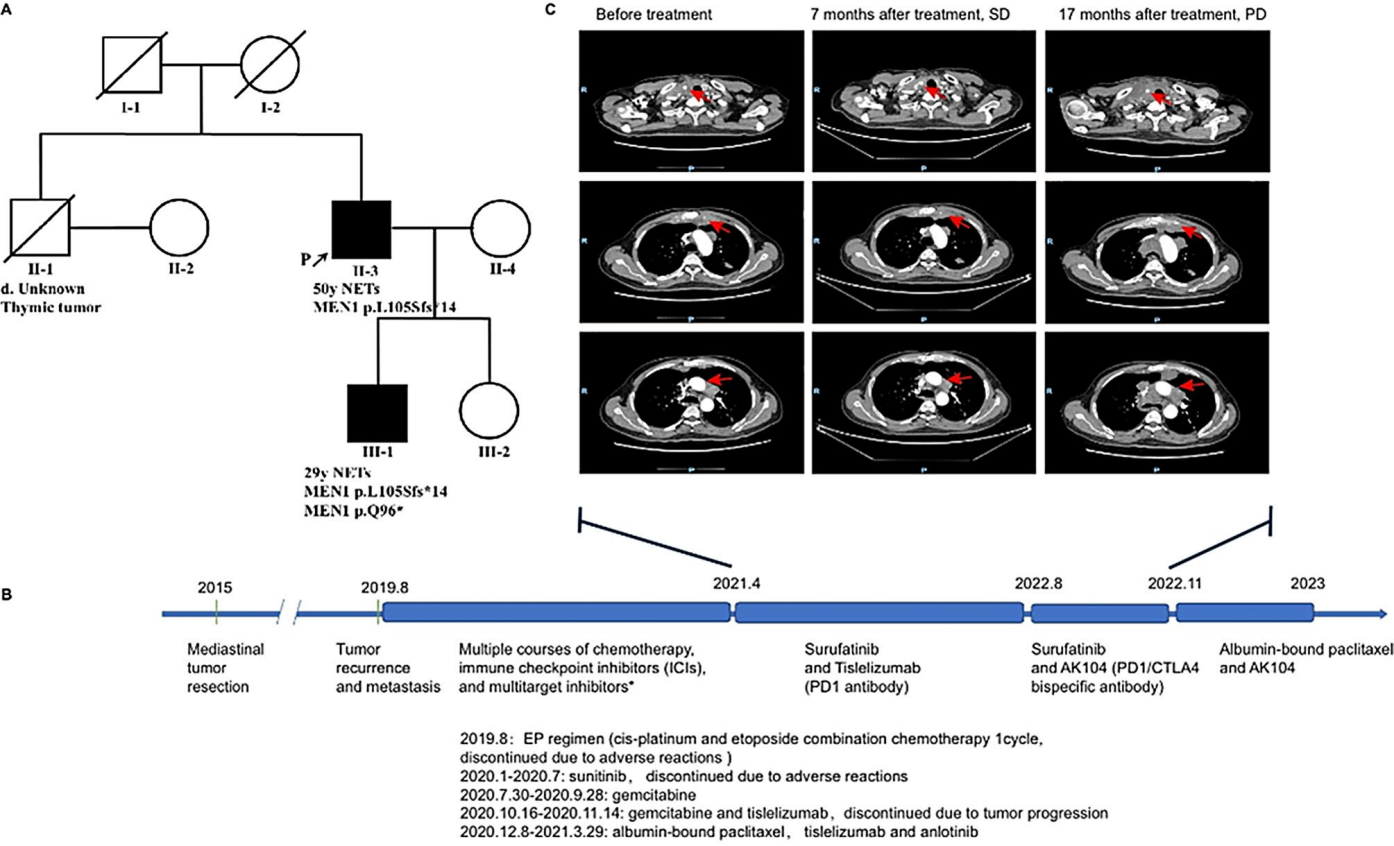
Pedigree Chart and treatment of the proband. **A**. Pedigree of a family with multiple endocrine neoplasia type 1 (MEN1). Solid symbols: clinically affected individuals with confirmed *MEN1* mutation; open symbols: family members without *MEN1* germline mutation; Slashed: decease; P: the proband; Patient 1; Ⅲ-1: the proband’s son, Patient 2 and Ⅱ-1: the brother of the proband. **B**. The therapeutic process of Patient 1. **C**. The axial view of contrast-enhanced computed tomography, Line 1: cervical lymph node lesions; Line 2: thymus lesions posterior to the sternum; and Line 3: mediastinal lymph node lesions

A 50-year-old man (proband, Ⅱ-3, Fig. 1A) was diagnosed with an atypical thymic carcinoid tumor and left adrenal cortical adenoma. His older brother had died of a thymic tumor. The proband’s 29-year-old son (Ⅲ-1; Fig. 1A) was also diagnosed with an atypical thymic carcinoid. This clustering of rare thymic neuroendocrine tumors across two generations prompted genetic testing for MEN1. Subsequent analysis confirmed two novel MEN1 variants. The detailed clinical courses of the proband and his son are described below.

**Fig. 2:**
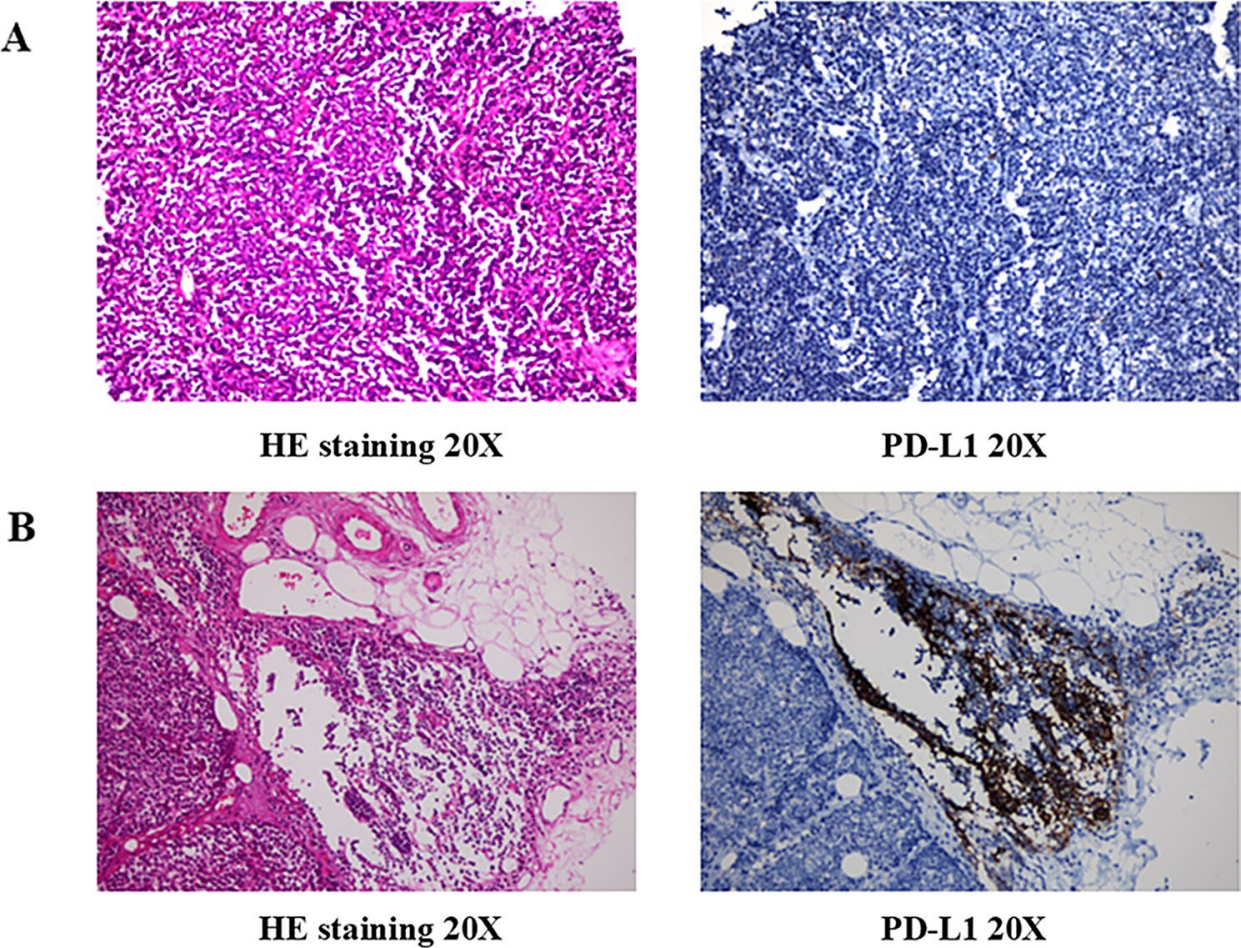
HE staining images and PD-1 expression images of the proband (patient 1), 20x magnification. (**A**) HE staining images and PD-1 expression images of the proband’s son (patient 2), 20x magnification. (**B**)

The proband was admitted to the local hospital presenting with a cough and chest pain of more than one month’s duration. The patient did not complain of dyspnea, fatigue, or ptosis. Contrast-enhanced computed tomography (CT) of the chest revealed the presence of an anterior mediastinal mass (2.0 cm × 3.2 cm) and a left adrenal gland mass (6.0 cm × 4.0 cm). In October 2015, the patient underwent mediastinal tumor resection at another hospital because of the enlargement of the anterior mediastinal mass (4.5 cm × 5.2 cm), as shown on CT. Pathological examination revealed R0 resection and in the presence of atypical thymic carcinoid tumor with macroscopic lesion necrosis and mitotic count of 6/10 high power field (HPF). IHC analyses revealed that the tumor cells were positive for markers such as creatine kinase (CK), chromogranin A (CgA), cluster of differentiation (CD)56, Syn, neuron-specific enolase, CK5/6, and CD117, with a Ki-67 proliferation index of 20%, whereas negative for the markers octamer-binding transcription factor 4, alpha-fetoprotein, CD30, placental alkaline phosphatase, human chorionic gonadotropin, P63, thyroid transcription factor 1, and CD99(Fig. 2A). Four years after the operation of the thymic tumor, the proband again visited the hospital with complaints of chest pain and cough (in 2019). CT suggested tumor recurrence in the anterior mediastinum (6.3 cm × 4.6 cm). The tumor had invaded the bilateral supraclavicular fossae, mediastinum, and hilum of the right lung, and the second anterior rib on the left side. The patient received multiple courses of chemotherapy, immune checkpoint inhibitors (ICIs), and small-molecule tyrosine kinase inhibitors(TKIs)for over 3 years. He was still alive in February 2023, with a good quality of life and regularly monitored for CT and serum neuron-specific enolase (NSE). The patient had received multi-treatment lines after relapse (Fig. 1B), among which the progression-free survival with the use of surufatinib was 17 months, as shown in Fig. 1C.

The proband had a history of lumbar disc herniation surgery in 1996 and was diagnosed with type II diabetes mellitus in 2000. Further, he was diagnosed with a left adrenal cortical adenoma in 2014, with a history of laparoscopic resection owing to enlargement of the left adrenal mass (8.0 cm × 7.5 cm). IHC staining of the cells revealed that they were weakly positive for Vimentin, with a Ki-67 proliferation index of approximately 2%, and negative for CK, α-inhibin, CD10, renal cell carcinoma, and CgA. No obvious necrosis was observed in the adenoma, with an HPF of 0/10. Additionally, the proband’s older brother had died owing to a thymic tumor.

The proband’s son was admitted to the hospital in March 2020 owing to an accidental finding of a thymic nodule on physical examination. At that time, he had no symptoms of dyspnea, fatigue, or ptosis. Subsequently, contrast-enhanced CT of the chest revealed a nodule (1.2 cm × 1.7 cm) in the right anterior mediastinum, with mild enlargement. The patient underwent robotic-assisted resection of the right anterior mediastinal nodule. Pathological examination revealed a grade R0 resection and the nodule to be an atypical thymic carcinoid without necrosis. The mitotic count was 10/10 HPF. Furthermore, IHC analyses revealed that the tumor cells were positive for CK, CgA, CD56, Syn, and CK19, with a Ki-67 index of 15%, but negative for terminal deoxynucleotidyl transferase, epithelial membrane antigen, carcinoembryonic antigen, CD1a, CD99, CD5, CD20, P40, and CD117(Fig. 2B). The patient recovered after surgery; no tumor recurrence was observed during regular review of CT and follow-up.

**Fig. 3:**
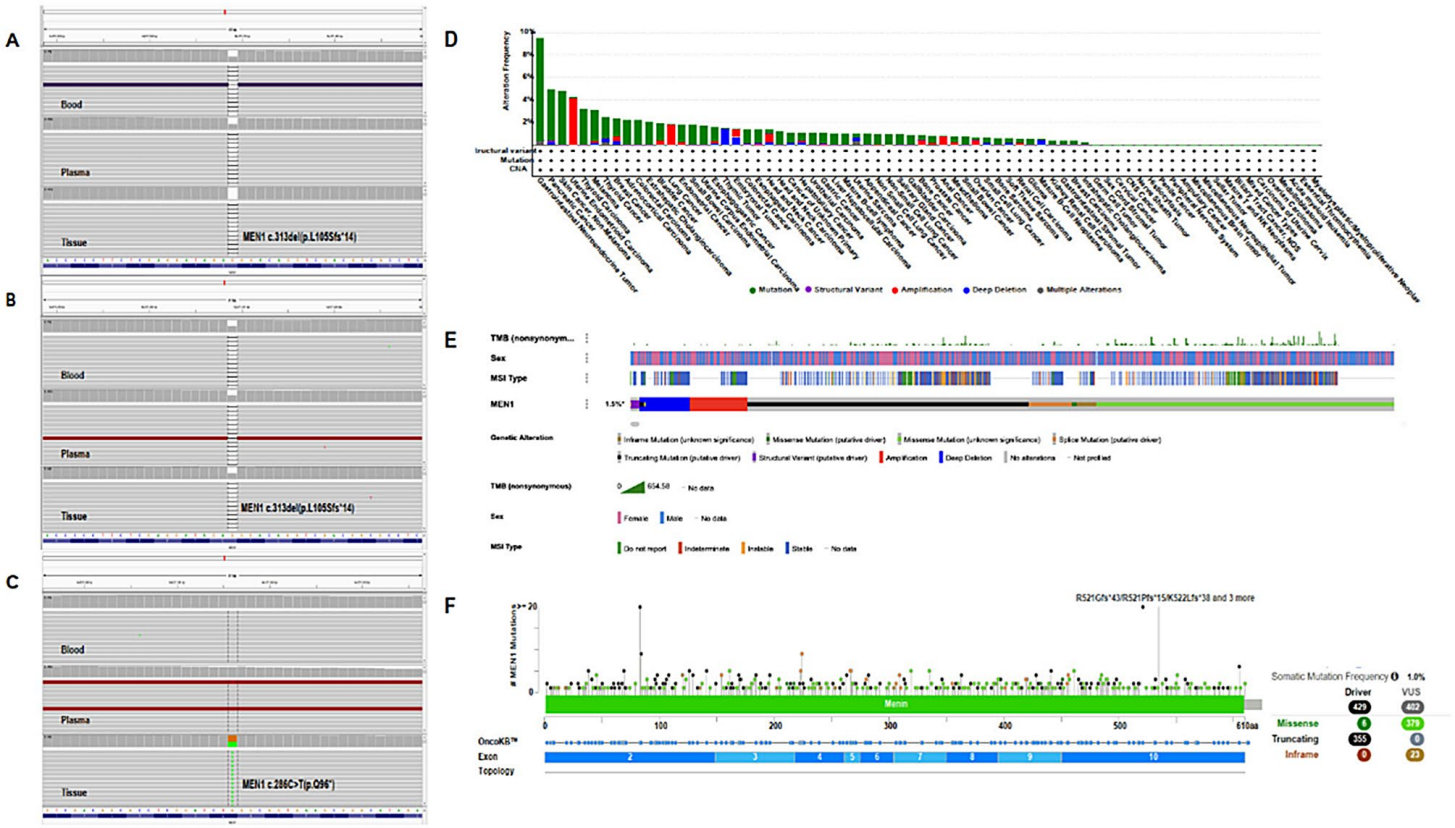
Integrative genomics viewer illustrating *MEN1* mutations and characteristics from cBioPortal. (**A**) Germline mutation c.313del (p.L105Sfs*14) in Patient (1) (**B**) Germline mutation c.313del (p.L105Sfs*14) in Patient (2) (**C**) Somatic mutation c.286 C > T (p.Q96*) in Patient 2. Pt.1: Patient 1, the proband, and Pt.2: Patient 2, the proband’s son. (**D**) Frequency of cancer types. (**E**) OncoPrint for patients with *MEN1* mutations presenting with clinical features. (**F**) Lollipop chart for driver genes of *MEN1* mutations and variants of undetermined significance

Considering the simultaneous discovery of thymus atypical carcinoids in both patients, surgical specimens and paired blood samples were collected for further testing. If the tumor proportion score was ≥ 1%, PD-L1 expression was positive; otherwise, it was negative. Paraffin-embedded tumor tissues and matched blood samples of the proband and his son were subjected to genomic profiling using the whole exome sequencing (WES) and 425 cancer-related gene panel (Geneseeq Technology Inc, Nanjing, China) based on next generation sequencing (NGS). Sequencing was performed using Illumina HiSeq sequencing platform (Fig. 3). The sequencing data from the tumor tissues of both the proband and the proband’s son have passed quality control. Results from both the 425 cancer-related gene panel and WES consistently reveal germline *MEN1* p.L105Sfs*14 mutations, and an additional somatic *MEN1* p.Q96* mutation was also detected in the proband’s son (Table 1). Based on clinical manifestations and laboratory examinations, the patients were diagnosed with MEN1.

**Table 1:** *MEN1* mutations and clinical test results of the two patients

	Pt.1	Pt.2
**MEN1 mutations**		
*MEN1* Germline mutation	*MEN1* c.313del(p.L105Sfs*14)	*MEN1* c.313del(p.L105Sfs*14)
*MEN1* somatic mutation		*MEN1* c.286 C > T(p.Q96*) VAF: 43.9%
**MSI analysis**		
No. of unstable MSI loci	9	13
MSI Score	0.1731	0.25
MSI Status	MSI-L	MSI-L
**HRD analysis**		
HRD Score	7	8
HRD Status	Negative	Negative
**TMB(Mutations/Mb)**	0.72/TMB-L	0.54/TMB-L
**PD-L1**		
TPS	Negative (< 1%)	Negative (< 1%)
CPS	Negative (< 1%)	Positive (1%)

## Discussion

The menin protein is structurally characterized by three tetratricopeptide repeat (TPR) motifs, flanked by N- and C-terminal helical bundles and capped by a three-stranded antiparallel β-sheet. Structural analysis of menin revealed the presence of a very large central cavity containing many hydrophobic and negatively charged AAs [9]. Although menin does not bind DNA directly, it plays a critical role in transcriptional regulation through its interactions with various regulatory proteins [9]. Therefore, mutations in *MEN1* will impair the stability of menin and its effects on protein–protein interactions. Furthermore, decreased interaction with menin may lead to increased cell proliferation.

In the present report, we described two generations of a Chinese family with thymic NETs. We used WES and 425 cancer-related gene panel to detect and identify two *MEN1* mutation sites (p.L105Sfs*14 and p.Q96*) that led to the development of pretruncated proteins. *MEN1* is encodes a 610-AA protein called menin. Menin plays a key role in cell division, genomic stability, and transcriptional regulation [22]. It is highly conserved in many species. The conservatism of AA analysis of the two novel *MEN1* variants in thymic NETs revealed that the AAs corresponding to these *MEN1* variants were highly conserved in multiple species.

To date, > 1300 germline and somatic mutations have been identified in *MEN1* [3, 8], resulting in MEN1-associated tumors. In the two patients with thymic NETs, the germline mutation was a heterogenic variant, *MEN1* Leu105 Serfs *14. This mutation caused the removal of the C base at position 313, leading to a shift in the genetic reading frame. This shift altered amino acid at 105 in the protein sequence and ultimately led to an early stop in the protein synthesis at amino acid 119. In addition to the germline *MEN1* mutation described above, we observed that the patients carried other common germline mutations (Table 2). On cBioPortal, the most common MEN1-associated tumors are gastrointestinal, pancreatic, skin, and other tumors. The most frequently occurring variant of the MEN1 gene is R521X. To the best of our knowledge, these two mutant variants (p.L105Sfs*14 and p.Q96*) have not been reported in relation to MEN1-associated thymic tumors.

**Fig. 4:**
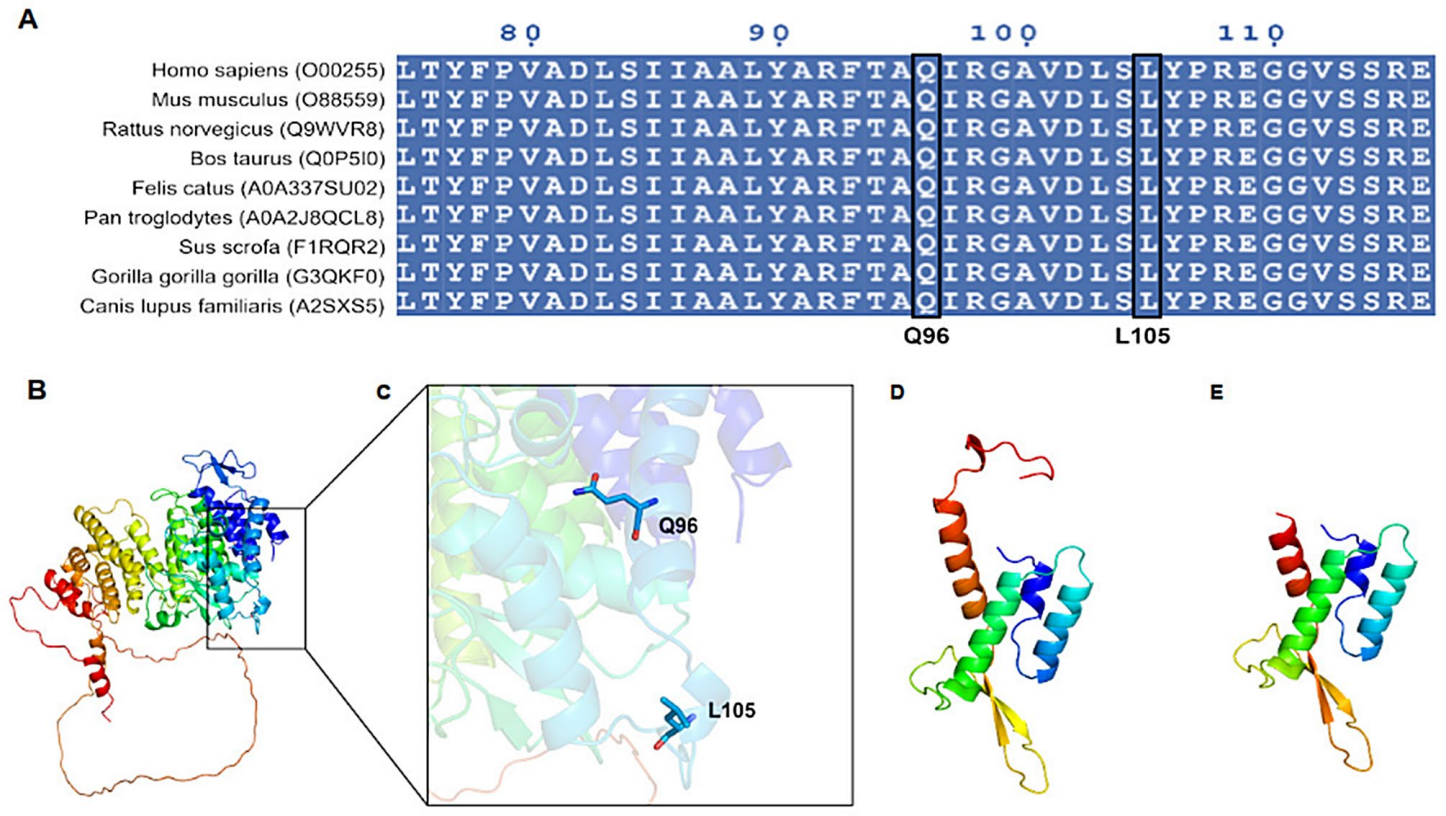
Conservatism analysis of AA and Modeled structure of menin. (**A**) The conservatism of 96 glutamine (Q96) affected by c.286 C > T and 105 leucine (L105) affected by c.313del across species. (**B**) Overall structure of the wild-type protein. (**C**) Two novel variants that led to protein truncation. (**D**) Mutated protein of the Q96 variant affected by the c.286 C > T mutation. (**E**) Mutated protein of the L105 variant affected by the c.313del mutation

A combined analysis of 10 pan-cancer studies revealed that the most frequent types of MEN1-associated tumors were gastrointestinal NETs, pancreatic cancer, and skin cancer (nonmelanoma), with thymus tumors ranking 18th (Fig. 3D). Among the 76,639 samples and 75,661 patients present in the combined analysis, only 902 patients and 915 samples harbored *MEN1* mutations. The OncoPrint map (Fig. 3E) indicated that there is no notable distribution of *MEN1* mutation based on TMB, microsatellite instability (MSI) type, and sex. The lollipop chart (Fig. 3F) suggested that in MEN1-associated tumors, missense mutations were more likely to be the variants of undetermined significance, whereas truncated mutations were more likely to be driver mutations. Among the driver genes of *MEN1* mutation, truncated mutation accounted for most of the proportion, with a frequency of 355, second only to missense mutations. Not only that, Bioinformatics analysis on cBioPortal revealed that the truncated mutations in the lollipop chart play an important role in MEN1-associated tumors. Truncation of its AA sequence will inevitably lead to the disruption of menin folding and, thus, loss of menin function. We downloaded the sequences of WT *MEN1* and two mutated *MEN1* variants and simulated the 3D structure of menin using AlphaFold2. We observed that both mutations lead to premature truncation of menin, affecting the cavity structure of menin, which may further affect its protein–protein interactions. Based on the bioinformatic analysis, 96 glutamine (Q96) and 105 leucine (L105) residues were found to be highly conserved in most species. (Fig. 4A). The WT and mutant models are shown in Fig. 4. The variant *MEN1* p.Q96* resulted in a stop codon. The sequence of co-occurrence was equivalent to the same sequence of *MEN1* p.Q96* mutation. Figure 4B-E demonstrates the WT and mutant models.

**Table 2:** Germline and somatic mutations detected by NGS of the two patients

Patient 1	Patient 2
**Germline mutation**	
*MEN1* c.313del(p.L105Sfs*14)	*MEN1* c.313del(p.L105Sfs*14)
*ARID1B* c.4490G > A(p.S1497N)	*EPAS1* c.1960G> (p.V654I)
*CDK12* c.2960 C > T(p.S987F)	*EPHA3* c.82 A > G(p.N28D)
*EPAS1* c.1960G> (p.V654I)	*PTCH1* c.3583_3584delACinsTT(p.T1195L)
*FAT1* c.10,226 C > T(p.T3409M)	
*IFNA6* c.140T> (P.I47T)	
*PTCH1* c.3584 C > T(p.T1195I)	
**Somatic mutation**	
*ASMT* c.361 > A(p.A121T)	*MEN1* c.286 C > T(p.Q96*)
*CHD1* c.3355 C > A(p.P1119T)	*AGK* c.370G > A(p.G124R)
*DOK6* c.880 A > T(p.M294L)	*CHEK1* c.676dupA(p.T226Nfs*19)
*EBF4* c.617G > A(p.R206H)	*FLG2* c.3319 C > T(p.P1107S)
*GABRA2* c.346_347del(p.L116Nfs*3)	*HPSE2* c. 1736del(p.Y579Lfs*74)
*GPRC5A* c.371G > A(p.R124Q)	*ITGB7* c. 1595G > A(p.G532D)
*LRRC34* c.314 A > G(p.E105G)	*LTN1* c.5423G > A(p.R1808H)
*MAP3K6* c.2575 A > G(p.M859V)	*MPRIP* c.504 + 1G > A(p.X168_splice)
*NHS* c.2009_2028del(p.L670Sfs*15)	*MUC19* c.11,128 C > G(p.P3710A)
*OR7D4* c.251G > A(p.S84N)	*MYO3A* c.2999G > A(p.R1000Q)
*PRSS33* c.376G > C(p.D126H)	*OR5H6* c.838T > A(p.S280T)
*QRFPR* c. 130G > A(p.A44T)	*RMDN1* c.289G > A(p.G97R)
*RP1L1* c.5626_5673del(p.A1876_E1891del)	*TCTE1* c.616 C > A(p.L2061)
*SEPTIN14* c.478G > A(p.V160M)	*TTN* c.91312G > A(p.D30438N)
*SETBP1* c.4691 C > G(p.P1564R)	*WDR81* c.3322 C > A(p.L1108M)
*SF1* c.541G > A(p.G181R)	*WNT8B* c.715 C > T(p.R239C)
*SLC16A11* c.1409 C > T(p.T470I)	*ZNF776* c.1493G > A(p.R498H)
*SLCO1A2* c. 1026C > A(p.Y342”)	*ZNF780A* c.1240 A > G(p.1414 V)
*SLIT1* c.2330 C > T(p.P777L)	
*SPTAN1* c.480 C > A(p.D160E)	
*SRP72* c. 1336 C > A(p.H446N)	
*TACC2* c.3805T > A(p.C1269S)	
*TNC* c.3805T > A(p.C1269S)	
*UPK3A* c.8 C > T(p.P3L)	

Both patients showed TMB-L, MSS, and low PD-L1 expression, supporting the results of WES and NGS. Furthermore, we found no significant increase in TMB on cBioPortal. The reason for this phenotypic profile exhibited by these tumors is that inactivation of the *MEN1* gene occurs predominantly through frameshift or nonsense mutations, which leads to loss-of-function of the menin protein rather than inducing genome-wide instability [23]. Moreover, *MEN1* inactivation does not involve *MMR* genes and thus does not induce MSI [24]. Furthermore, MEN1-associated tumors typically lack robust immune cell infiltration and a pro-inflammatory tumor microenvironment [25]. The characteristics of TMB and PD-L1 expression in MEN1-associated NETs suggest that immune checkpoint blockers (ICBs) may be less effective in these patients [26], therefore, combination therapeutic strategies should be considered [27]. These preliminary observations warrant validation in larger patient cohorts.

Although thymic tumors are relatively rare among *MEN1* mutation-positive tumors, they ranked 18th on cBioPortal. However, studies have reported that thymic tumors are the second most common cause of death in patients with MEN1 after pancreatic NETs [2, 28]. Surgery was the preferred treatment option for early-stage thymic NETs, and chemotherapy is recommended for patients with NETs in the locally advanced and metastasis stages. Both patients underwent surgical resection at an early stage of disease, the proband received multiple courses of chemotherapy, ICIs, and TKIs after tumor recurrence. Surufatinib, a multi-kinase inhibitor targeting VEGFR1-3, FGFR1 and CSF-1R [29], showed significant PFS benefit in pancreatic and non-pancreatic NETs in two phase III trials (SANET-ep/p) [30] and was approved by China’s NMPA for non-pancreatic NETs in 2020. As of the last follow-up in September 2022, the proband had achieved a progression-free survival (PFS) of 17 months. In the phase III clinical trial of SANET-ep, non-pancreatic NET patients treated with surufatinib experienced a median PFS of 9.2 months, compared to 3.8 months for patients receiving placebo [31]. Currently, the proband still maintains a good quality of life.

MEN1 is an autosomal dominant disorder with first-degree relatives having a 50% risk of disease development and usually identifiable by *MEN1* mutation analysis. All patients with MEN1-related tumours should undergo genetic testing. Family screening should be done after confirming the *MEN1* mutation [32, 33]. The proband was simultaneously diagnosed with two endocrine tumors at 49 years of age, but no genetic testing was performed and therefore no genetic counselling was offered. Subsequent WES revealed a germline mutation in *MEN1*, consistent with the diagnosis of MEN1. In addition to the germline mutation in *MEN1*, p.L105Sfs*14, a somatic mutation in *MEN1*, i.e., p.Q96*(This means that there was a change in the DNA sequence at position 286 where the base C was replaced by T. This caused a stop codon to be formed and ultimately ended the translation process early at amino acid 96.), was detected in the son of the proband, at 29 years of age. These two mutation sites are not on the same DNA strand, indicating that the gene may have undergone biallelic gene inactivation. This finding is consistent with Knudson’s “two-hit hypothesis”. This suggests that two-hit may lead to early tumor onset. Therefore, our case report highlights the importance of the early diagnosis and screening of germline mutations in the families of patients with MEN1. Unfortunately, the archival specimens were formalin-fixed and paraffin-embedded(FFPE) and subjected to prolonged storage. This may have resulted in protein degradation, precluding reliable protein-based assays with current methodologies. In some of the literature, the “two-hit hypothesis” can be verified using heterozygous genetically engineered mouse models, which we cannot verify at this time due to limited funding and time. We look forward to verifying this in future experiments.

In summary, we described the case of a family with MEN1-associated thymic NETs, which resulted in premature truncation of the protein. The conservation of Q96 and L105 AA residues in different species indirectly suggests the pathogenicity of the Q96 and L105 variants. In addition, simulated protein structures suggest that both variants lead to the production of truncated menin protein, possibly affecting protein function. Further experiments are warranted to verify whether these variants cause a loss of protein function. We treated one of the progenitors with surufatinib, chemotherapy and ICB and achieved good clinical results.

## Data Availability

The content and data related to patients in this article have been obtained with the consent of the patients, who agree to share them with the publishing house. We have removed personal information such as the patients’ names, genders, identification numbers, and other information that may identify the patients to ensure their privacy. The original contributions presented in the study are included in the article. Further inquiries can be directed to the corresponding author.
